# Pneumatization of the zygomatic process of temporal bone on computed tomograms

**DOI:** 10.3205/iprs000095

**Published:** 2016-06-14

**Authors:** Reinhard E. Friedrich, Liska Viezens, Ulrich Grzyska

**Affiliations:** 1 Oral and Craniomaxillofacial Surgery, Eppendorf University Hospital, University of Hamburg, Germany; 2 Neuroradiology, Eppendorf University Hospital, University of Hamburg, Germany

**Keywords:** pneumatization of bone, zygomatic air cell, computed tomography, glenoid fossa, zygomatic arch, temporal bone

## Abstract

**Purpose:** Zygomatic air cells (ZAC) are a variant of temporal bone pneumatization that needs no treatment. However, ZAC can have an impact on surgical procedures in the temporo-mandibular joint region. Recent reports suggest that computed tomography will disclose more ZAC than can be diagnosed on panoramic radiography. The aim of this study was to analyze ZAC prevalence on CT in a population that was not pre-selected by admission to a dental clinic. Furthermore, an extensive literature review was performed to assess the prevalence of ZAC and to address the impact of imaging technique on the definition of the item.

**Material and methods:** Digitalized cranial CTs of 2007 patients were retrospectively analyzed. The Frankfort horizontal was used to define a ZAC on sagittal CTs.

**Results:** In this study group, 806 were female (40.16%) and 1,201 were male (59.84%). Mean age was 49.96 years in the whole group (female: 55.83 years, male: 46.01 years). A ZAC was diagnosed in 152 patients (female: 66, male: 86). Unilateral ZAC surpasses bilateral findings (115 vs. 37 patients). ZAC were diagnosed in children 5 years of age and older. Sectional imaging techniques show a better visualization of the region of interest. However, presently an increase of ZAC prevalence attributable to imaging technique cannot conclusively be derived from the current literature. The normal finding of a ZAC on radiograms is a sharply defined homogenous transparent lesion restricted to the zygomatic process of the temporal bone that has no volume effect on the shape of the process.

**Conclusion:** ZAC is an anatomical variant of the temporal bone that has come into focus of maxillofacial radiology due to its noticeable aspect on panoramic radiograms. The harmless variant can be expected in about one in thirteen individuals undergoing facial radiology. Panoramic radiograms appear to be sufficient to present ZAC of relevant size. However, in preparation for surgical procedures affecting the articular eminence the application of sectional images is recommended.

## Zusammenfassung

**Ziel:** Pneumatisationen des Jochbogens [Zygomatic air cells (ZAC)] stellen eine anatomische Variante der Pneumatisationen des Schläfenbeins dar, die keiner Behandlung bedürfen. Allerdings können ZAC die Planung und Durchführung chirurgischer Maßnahmen der Kiefergelenksregion beeinflussen, z.B. bei der Weiterleitung von Infektionen. Aktuelle Berichte legen die Schlussfolgerung nahe, dass die Computertomographie (CT) häufiger ZAC abbildet, als dies mit der in der Erstdiagnostik der Kiefer häufig gebräuchlichen Panoramaschichtaufnahme (PSA) gelingt. Das Ziel dieser Untersuchung war die Bestimmung der Prävalenz von ZAC auf CT der Temporalregion bei einer Untersuchungsgruppe, die nicht durch Zuweisung an eine Zahnklinik vorselektioniert gewesen ist. Zusätzlich wurde eine ausgedehnte Literaturrecherche durchgeführt, um die Prävalenz der ZAC einzuschätzen und den Einfluss der Untersuchungstechnik auf die Definition der Entität zu bestimmen.

**Material und Methoden:** Digitalisierte kraniale CTs von 2007 Patienten wurden retrospektiv analysiert. Die Frankfurter Horizontale wurde benutzt, um ZAC auf sagittalen CT zu definieren.

**Ergebnisse:** In der Untersuchungsgruppe befanden sich 806 weibliche (40.16%) und 1201 männliche Patienten (59.84%). Der Mittelwert des Lebensalters der Gesamtgruppe war 49,96 Jahre (weiblich: 55,83 Jahre, männlich: 46,01 Jahre). Ein ZAC wurde diagnostiziert bei 152 Patienten (weiblich: 66, männlich: 86). Die Anzahl unilateraler ZAC übertraf die bilateraler Befunde (115 vs. 37 Patienten). ZAC wurden bereits bei Kindern im Alter von 5 Jahren und etwas älter befundet. Die CT bildet die interessierende Region eindeutig anschaulicher ab. Allerdings kann die Auswertung der aktuellen Literatur nicht bestätigen, dass die Bevorzugung der CT als Untersuchungstechnik häufiger zu einem Nachweis von ZAC führt als dies in der PSA der Fall wäre. Der Normalbefund einer ZAC ist ein scharf begrenzter homogen strahlentransparenter Bezirk ohne Einfluss auf die Form des Knochens und beschränkt auf den Jochbogenfortsatz des Schläfenbeins.

**Schlussfolgerung:** ZAC sind anatomische Varianten des Schläfenbeins, die in den Fokus der maxillofazialen Radiologie geraten sind, weil sie auf radiologischen Routineaufnahmen (PSA) einen auffälligen Befund darstellen können. Die harmlose anatomische Variante kann in ca. einem von dreizehn Patienten erwartet werden, deren Region im Strahlengang einer Schichtbildtechnik abgebildet wird. Die PSA ist ganz offensichtlich ausreichend, um relevante ZAC orientierend abzubilden. Allerdings sollte in der Vorbereitung operativer Eingriffe an der Eminentia articularis die Verwendung von Schichtaufnahme-Techniken bevorzugt werden, die diese Region in mehreren Ebenen abbilden.

## Introduction

**Figure 1:**
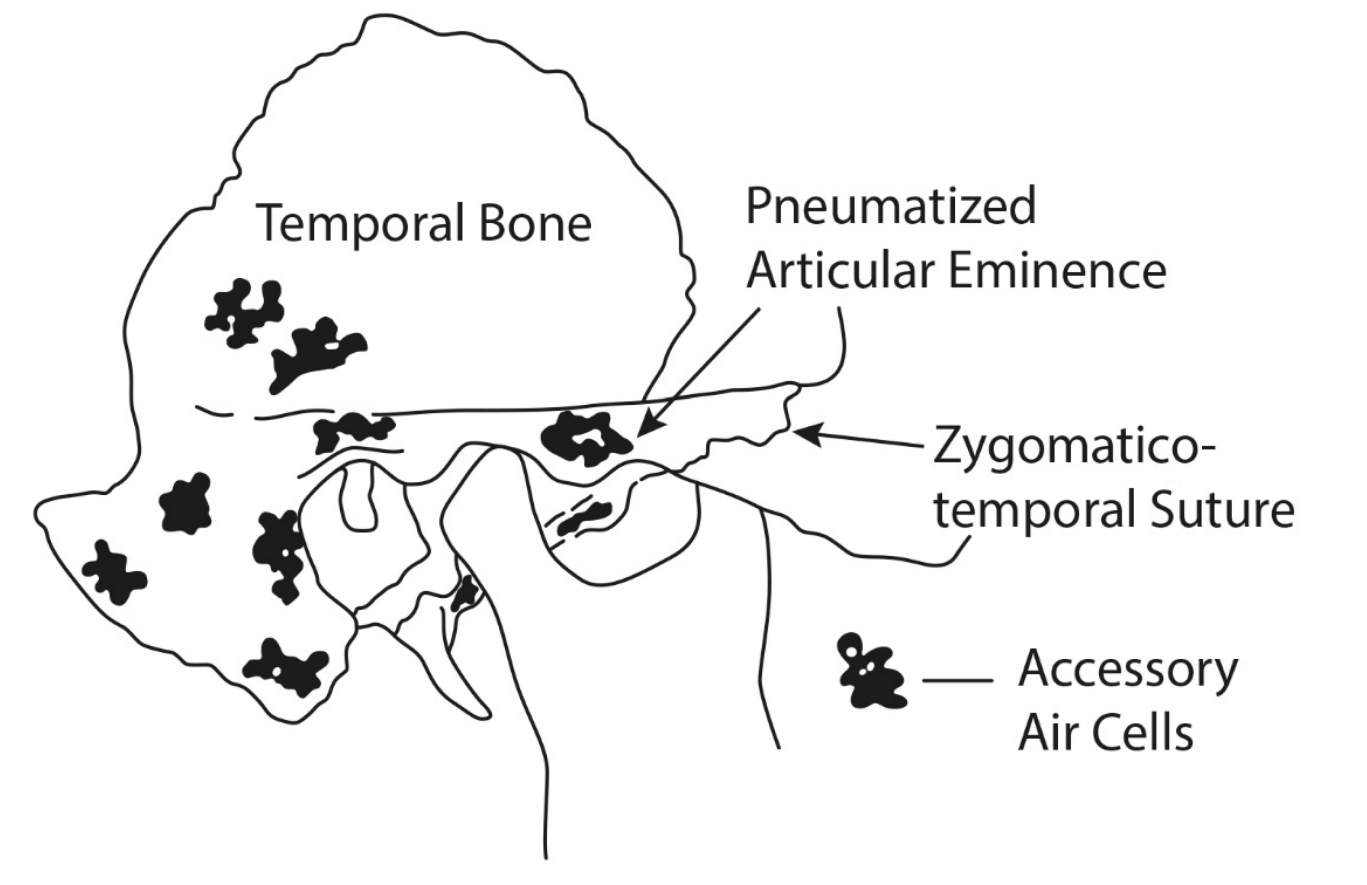
Location of zygomatic air cells according to Tremble (1934) [61]

The development of the skull is accompanied by multiple pneumatizations at different locations [39]. Some of these pneumatizations are air-filled cavities completely enclosed by bone, also known as air cells (AC) [12]. The best known of these are the mastoid AC in the temporal bone. These AC can cause severe problems due to pathologies, such as tumor or infection of the mastoid, and their consecutive transmission in adjacent regions, including the zygoma [12], [18], [68]. Origin and spread of temporal bone pathologies make clear the preserved connection between the disseminated AC within the temporal bone and the mastoid antrum (Figure 1 (Fig. 1)). Some locations of AC inside the temporal bone can be expected as a characteristic feature but others only occur occasionally [54], [61]. Indeed, the individual distribution of AC varies considerably and correlates poorly with age and gender [65]. Tremble classified 10 regions of AC in the temporal bone with particular reference to illustrate pathways of mastoid infection [61]. One of the temporal bone’s AC locations is the root of the zygomatic process forming the glenoid fossa [18]. AC of the zygomatic process can occasionally even extend into the arch [39], [61]. Tyndall and Matteson re-addressed the phenomenon of the pneumatized articular eminence from a radiological point of view. They specified it as a radiolucent, asymptomatic ‘defect’ in the zygomatic process of the temporal bone on panoramic radiographs showing a similar appearance as the mastoid AC. The ‘defect’ optionally approximates, according to their statement, up to the temporo-zygomatic suture, but never crosses this junction. In addition, they also reaffirmed the zygoma as of normal shape without any kind of altered outline or destruction [62].

Pneumatization of the temporal bone’s zygomatic process is a variant of human anatomy that needs no treatment [49] but is a relevant finding in the planning of temporo-mandibular joint (TMJ) surgery [26]. During TMJ operations a pneumatized fossa can hamper surgical procedures [7], [50], [67]. Indeed the alternative surgical treatment protocol was proposed for recurrent mandibular dislocation in patients with zygomatic AC (ZAC) [7]. Furthermore, extensive ZAC can be associated with TMJ dysfunction [25] and ZAC were surprisingly frequently diagnosed in patients with TMJ problems [22]. In addition, some surgical procedures on malar osteoplasty have to consider the internal structure of the zygomatic arch [6], [59].

Nowadays, zygomatic abscess in the course of temporal bone infection is rare [61], [66], but knowledge about this way of infection is mandatory in differential diagnosis of inflammatory disease in the lateral skull base [36].

The aim of this study was to determine the frequency and topography of zygomatic air cells with respect to age and gender on computed tomograms and to compare our findings with results from other studies with reference to the impact of imaging method on the reported frequency of this osseous variant.

## Material and method

### Assortment of images

The temporal bones were reviewed for zygomatic air cells in 2,007 patients who were subjected to regular skull computed tomography (CT) at the University Hospital Hamburg-Eppendorf. The period of the studied CT images ranges from 2009 to 2013. CT images of patients that were incomplete or had blurring, had been excluded from the patient population. Patients with a history of maxillofacial trauma or temporal bone pathology were excluded from evaluation as were patients with a known history of skull development alterations.

### CT scanners

The CT scanners used for the study were the model Philips MX 8000 IDT 16, Philips Brilliance Brilliance iCT 64 and 256 (Philips Healthcare, Best, The Netherlands). The device MX 8000 IDT 16 is a 16 line coil computed tomography. The Brilliance 64 is also a coil CT scanner, which however, takes up 64 lines. The Brilliance ICT 256 writes up to 256 lines in the longitudinal axis. The tube voltage was 120 kV, the slice thickness 3 mm. After the pictures were taken, they were stored and edited in the Picture Archiving and Communication System (PACS IW, General Electric Healthcare, Milwaukee, USA).

### Analysis of images

Digitalized skull CT images were screened for ZAC on a diagnostic monitor (Department of Radiology, UKE). Any pneumatization in following regions was recorded: the articular eminence, the articular tubercle or the zygoma cranial or anterior to the temporal fossa. The images were viewed in the sagittal plane, as well as in the axial plane. ZAC was defined using the Frankfort horizontal. On sagittal images, the dorsal definition of the Frankfort horizontal was the most cranial portion of the glenoid fossa and anterior definition the most cranial point of the infraorbital rim on the same CT sectional image. All patients were diagnosed as individuals with ZAC, in whom well defined air-equivalent disruptions of bone contour within the zygomatic arch were visible inferior to the Frankfort horizontal plane. This definition was chosen in order to avoid inaccurate topographical signs of the temporal bone. 

### Statistical analysis

For the statistical analysis of own findings, gender, age and location of the ZAC was collected. For data analysis, metric variables with mean value and standard deviation as well as categorical variables with the absolute and relative frequencies were determined. For the age dependency a logistical regression was computed, the dependent variable was formed by the presence of ZAC. Age was the independent variable. 

For the analysis of gender dependency, chi-square test was used (p-values are reported). 

A p-value under 5% indicates statistical significance. All tests are performed two sided. All analyses were computed with IBM SPSS™ (Version 21.0., IBM Corp., Armonk, NY) and PSPP (psppire 0.8.4, GNU project).

### Review

Mesh terms ‘pneumatization’, ‘zygomatic air cell’, ‘zygomatic air cell defect’, ‘pneumatized articular eminence’, combined with ‘computed tomography’, ‘cone beam computed tomography (CBCT)’, ‘panoramic radiograph’ and combinations thereof were searched in PubMed database. In addition, these terms were also used for a Google™ search of references not provided by PubMed.

### Ethics

The investigation was approved by the University Hospital Authority Board as a prerequisite to achieve the doctoral degree in medicine (LV). All patients had given informed consent for scientific investigation of medical findings. For this study, no ethics vote was needed.

## Results

ZAC is a radiotranslucent finding restricted to the temporal part of the zygomatic arch. The cavity is a distinct osseous lesion with well demarcated borders. In no instance the cortical layer surrounding the ZAC is interrupted. CT signals of the cavity’s internal structure are equivalent to air. Focal inhomogeneities projected into the lumen in a single section are in every case identifiable on perpendicular sections as the transition of the bone into the cavity. Therefore, radiopacities inside the cavity are judged to be no physiological finding of ZAC on CT.

### Total group

Out of 2,007 patients 806 were female (40.16%) and 1,201 (59.84%) male. The mean age of the patients was 49.96 years (SD=22.03 years, rang 0 to 102 years). The mean age of women was 55.83 years (SD=22.82 years, range 3 to 102 years). The mean age of the men was 46.01 years (SD=20.66 years, range 0 to 100 years). 

### Age distribution of patients with ZAC 

One hundred and fifty-two patients (7.57%) of the study had pneumatizations located within the articular eminence, the articular tubercle and in the zygomatic process of the temporal bone. In all these cases it was stated that once a pneumatization occurs in the zygomatic process it also extends over the glenoid fossa, in other words: the formation of ZAC are dependent on a pneumatized glenoid fossa. 

Sixty-six patients with ZAC (8.19%) were female, and 86 (7.16%) male. The mean age of patients with ZAC was 45.12 years (SD=20.38 years, range 5 to 90 years). Mean age of women with ZAC was 47.53 years (SD=20.51 years, range 5 to 90 years) and the mean age of men was 43.27 years (SD=20.20 years, range of 6 to 90 years). 

### Laterality of ZAC

**Figure 2:**
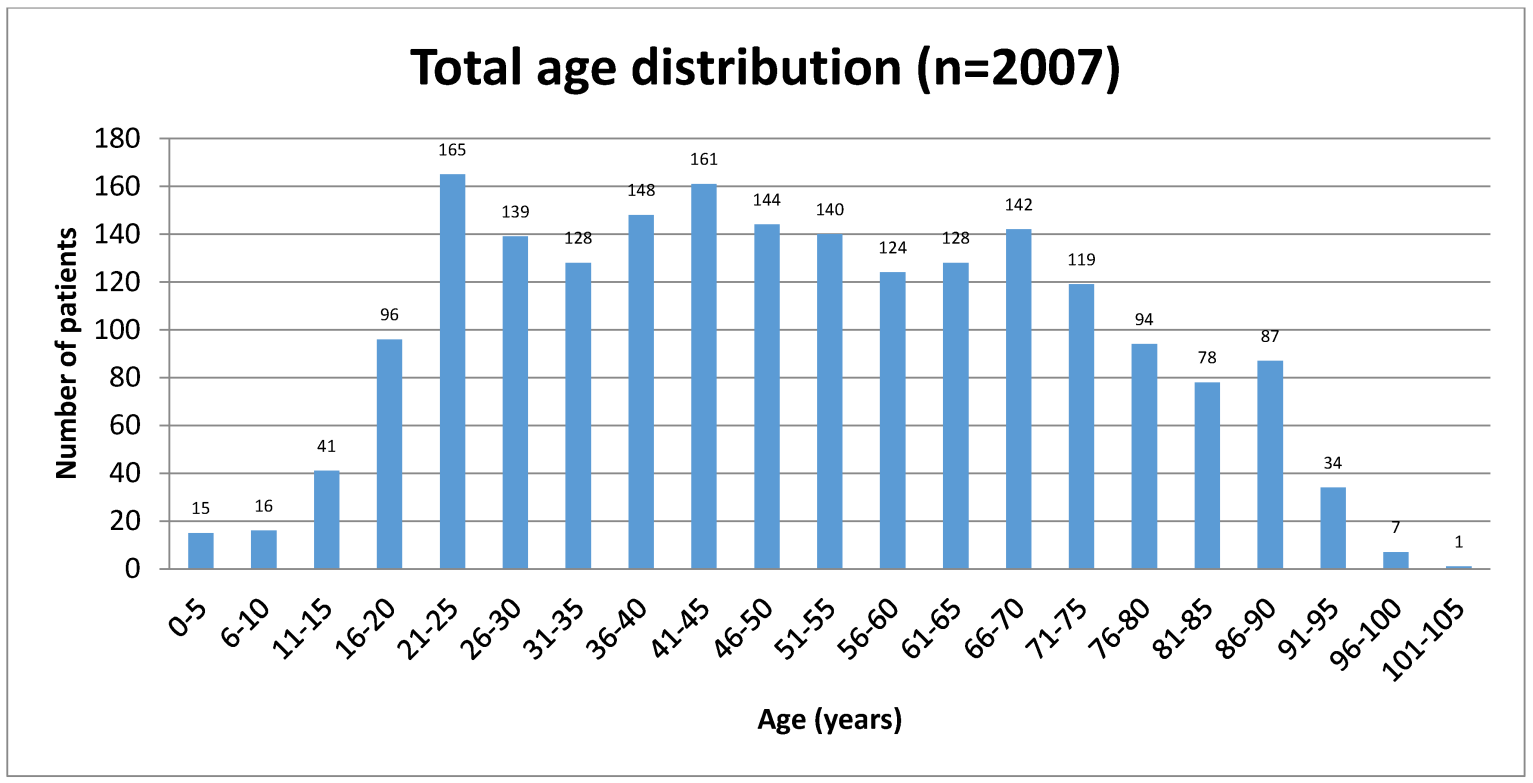
Age distribution of patients studied for zygomatic air cells

**Figure 3:**
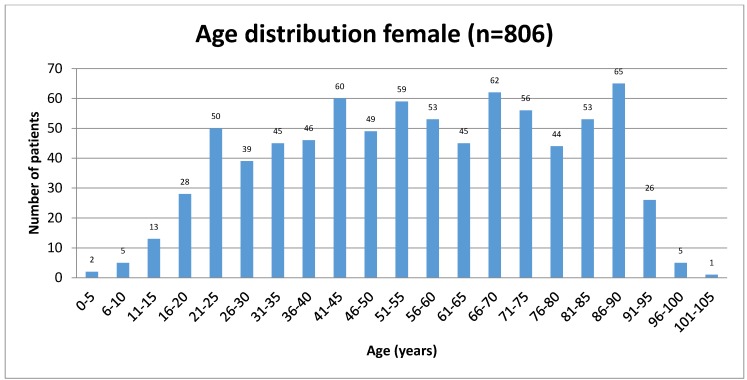
Age distribution of female patients studied for zygomatic air cells

**Figure 4:**
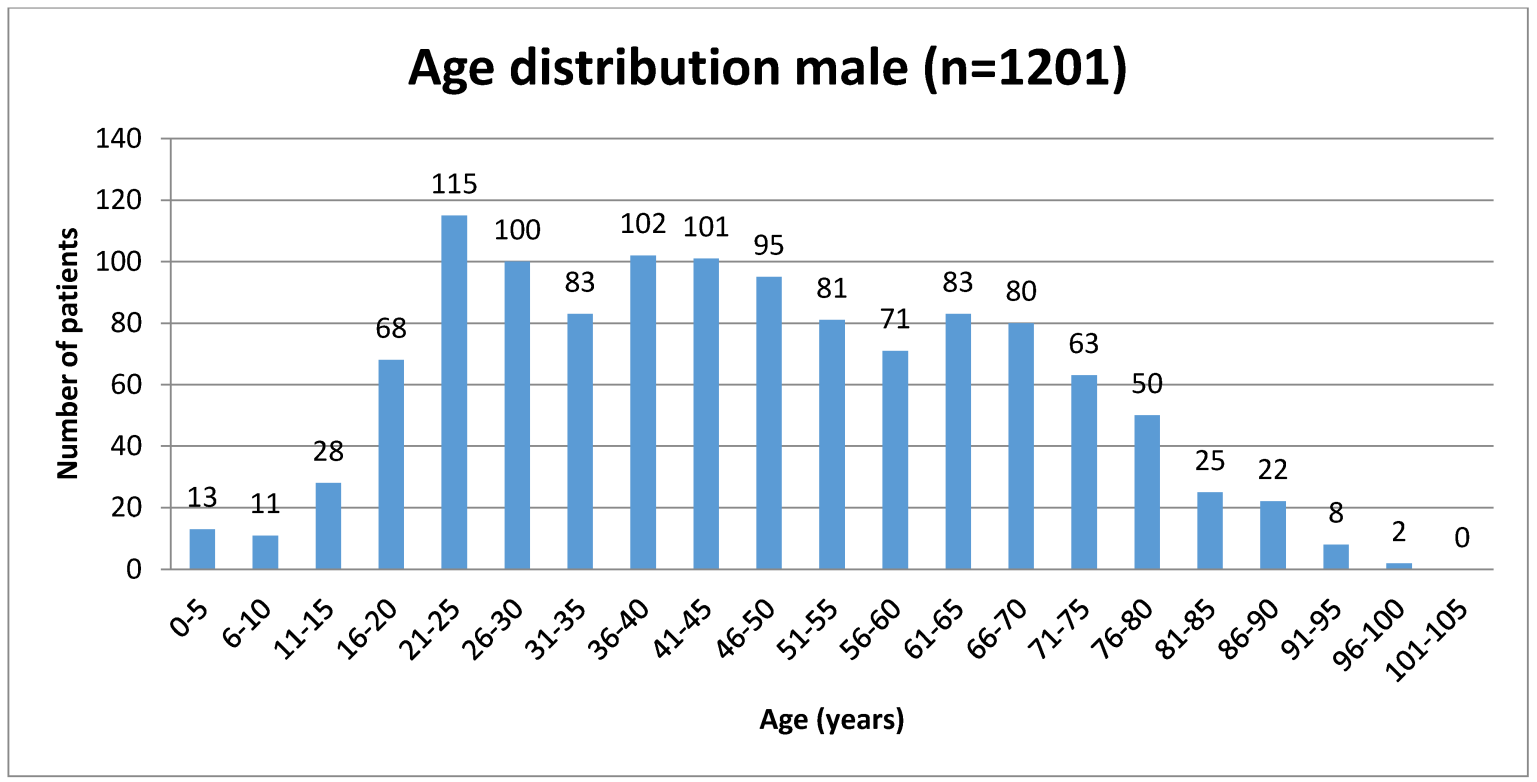
Age distribution of male patients studied for zygomatic air cells

**Figure 5:**
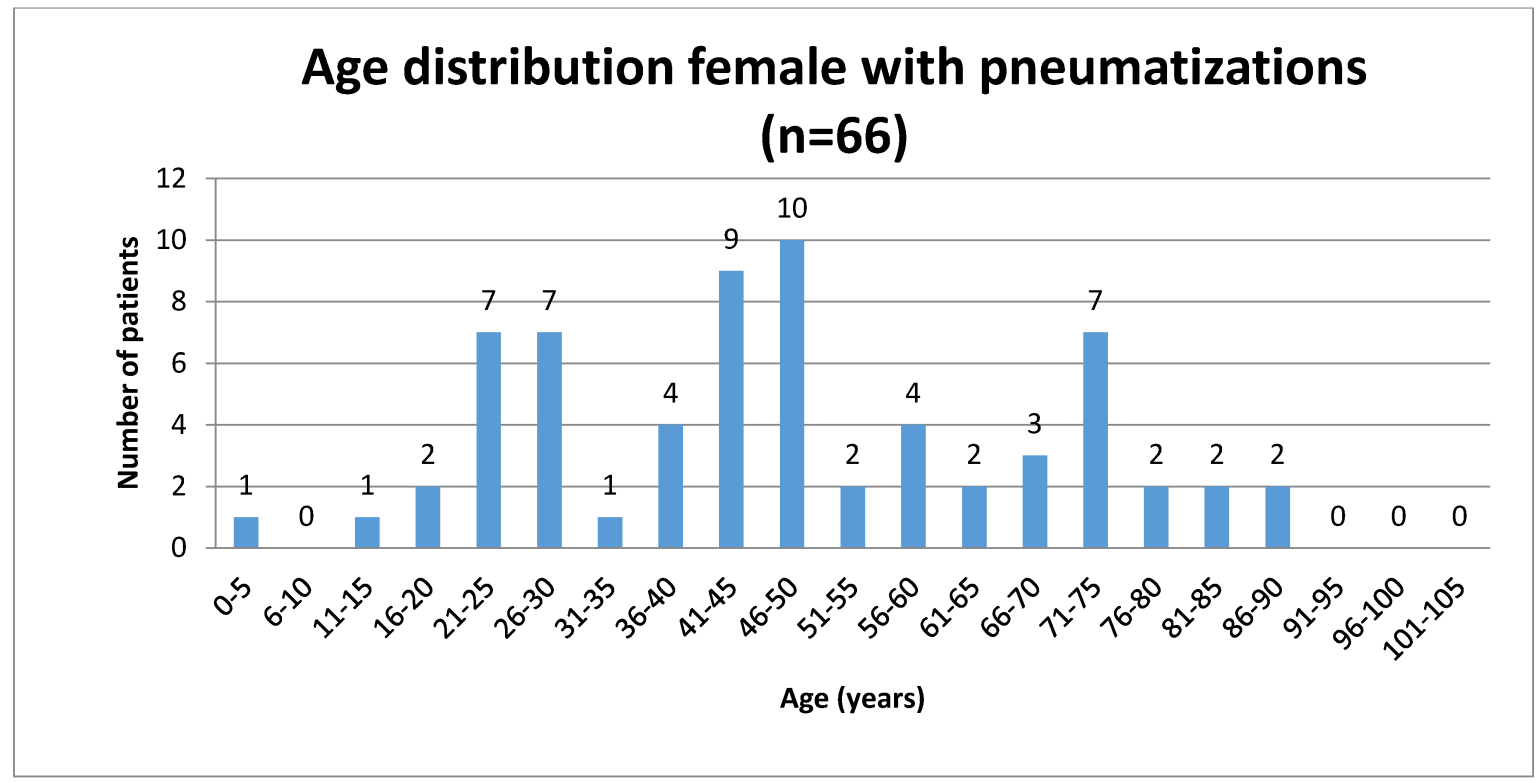
Age distribution of female patients studied with zygomatic air cells

**Figure 6:**
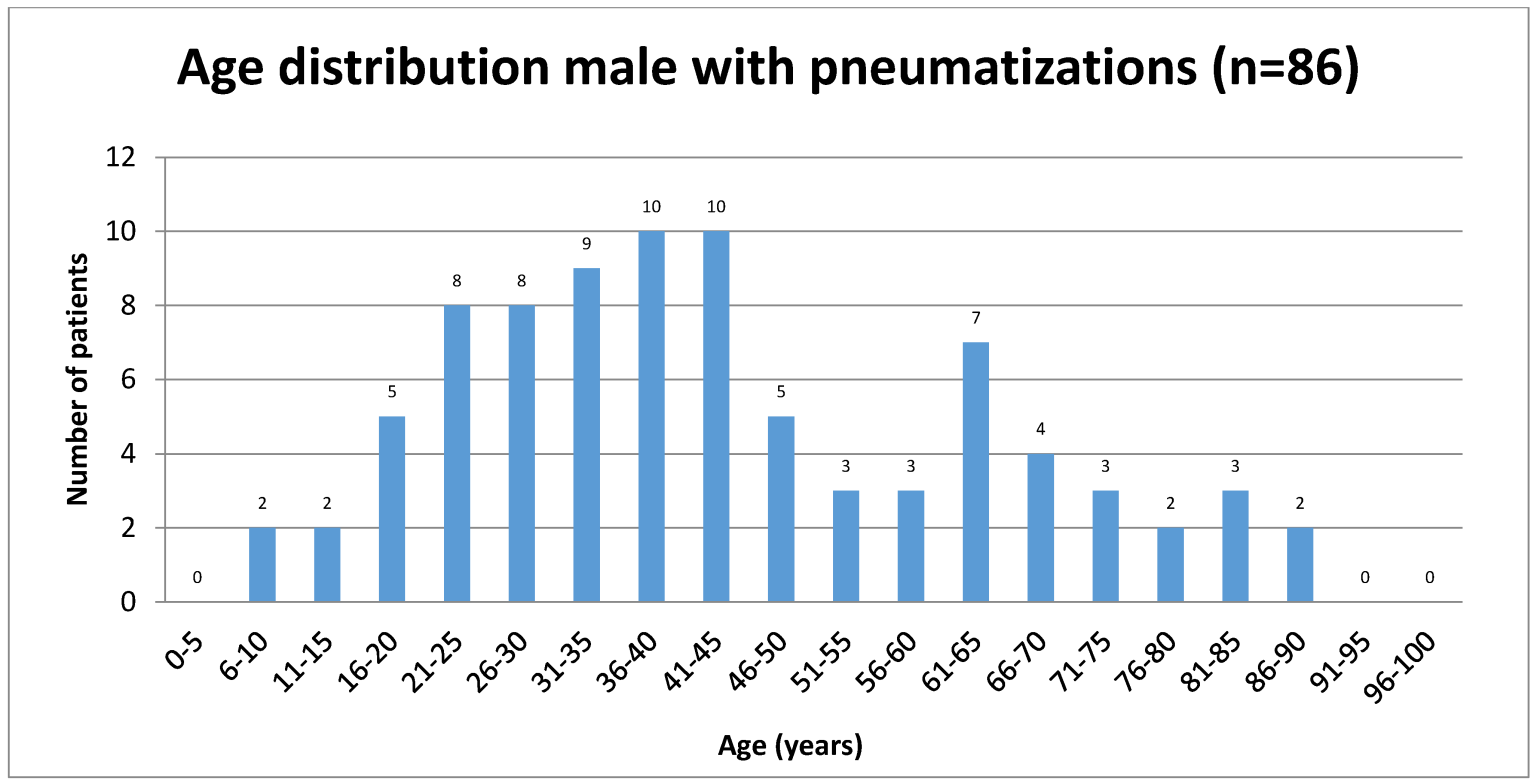
Age distribution of male patients studied with zygomatic air cells

**Figure 7:**
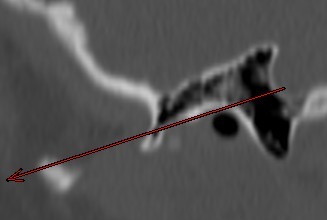
Zygomatic air cell in a 5-year-old patient

**Figure 8:**
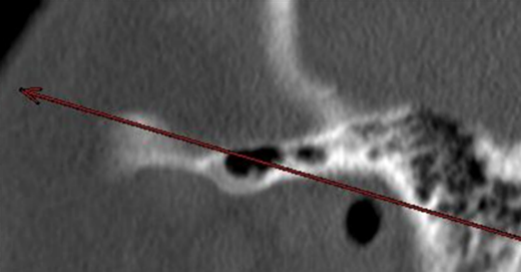
Zygomatic air cell in a 90-year-old patient

ZAC occurred in 115 (5.73%) patients unilaterally and in 37 (1.84%) ambilateral. On the left side 66 patients had ZAC, (females: 26 (39.39%), males 40 (60.61%)). Forty-nine cases of ZAC occurred on the right side (females: 23 (46.94%), males: 26 (53.06%)). Bilateral ZAC of 37 patients was 17 (45.95%) in females and 20 (54.05%) in males. Results are summarized in Table 1 (Tab. 1) and Table 2 (Tab. 2) and illustrated in Figure 2 (Fig. 2), Figure 3 (Fig. 3), Figure 4 (Fig. 4), Figure 5 (Fig. 5), Figure 6 (Fig. 6), Figure 7 (Fig. 7) and Figure 8 (Fig. 8).

The chi-square test showed no significant correlation between the parameter ‘gender’ and ‘age’ and the prevalence of pneumatization (p<0.05). However, p-value verges on the significant range for the parameter ‘age’, indicating only a trend. The probability of occurrence of pneumatizations in adulthood decreases by 1% with each year the age increases. 

### Literature review

Publications were selected providing data on ZAC prevalence that were based on larger series of radiographs. In order to illustrate the impact of imaging technique on the prevalence rate, studies that had analyzed panoramic radiographs are reproduced separately from studies who evaluated multiple sectional images (Table 3 (Tab. 3) and Table 4 (Tab. 4)). The prevalence of ZAC on panoramic radiographs of the published studies is usually less than 5% excepting 2 recent reports from Iran and India that presented prevalence higher than 5%. Furthermore, in one study with symptomatic TMJ the ZAC prevalence was exceedingly high (Table 3 (Tab. 3)). In several publications the prevalence of ZAC is definitely higher on sectional cross sectional images than on panoramic radiographs (Table 4 (Tab. 4)). However, 2 recent studies from Turkey and Brazil reveal ZAC prevalence on CBCT in the range of panoramic radiography derived results (Table 4 (Tab. 4)). Mean value of ZAC prevalence depending on imaging technique is only slightly higher in computed tomography studies (9.7% vs. 7.8%). Therefore, the dependence of ZAC detection on the resolution of the applied imaging technique, that is obvious on the first glance, cannot be confirmed with respect to all currently published radiological studies. Indeed, the number of authors is extremely limited who are investigating ZAC to allow general recommendations for preferring an imaging method. Credit should be given to Andersen [1] who was very likely the first investigator publishing a large study on ZAC prevalence on rotational panoramic tomograms. This author reported on children aged 10 years or older showing ZAC by this method [1].

## Discussion

This study shows that about one in thirteen patients will show a pneumatization of the zygomatic arch on CT and is quite a frequent radiological finding. Furthermore, this variant of temporal bone pneumatization occurred not independently but in all our cases was associated with a pneumatized glenoid fossa, as already noted by the authors of the first CT-based study on ZAC prevalence [20]. However, these authors did not apply the Frankfort horizontal as a defining landmark to distinguish ZAC of the zygomatic process from an extended pneumatized glenoid fossa.

Although the AC were predominantly diagnosed in adults, this study reveals foci of a pneumatized zygomatic arch even in children 5 years of age. This finding is in line with basic morphological studies on the very early onset of pneumatization in the human temporal bone [68].

Up to now the definition of what region should be recognized a preferential origin of the temporal bone’s pneumatization relies on a classification popularized by Tremble [61] (Figure 1 (Fig. 1)). Synonymous descriptions of this radiological findings are (zygomatic) AC [12], [57], cyst-like pneumatization of articular tubercle [1], ZACD [56], [63], pneumatized articular eminence/tubercle or zygomatic pneumatization [32], [37], [47]. 

In 1985 Tyndall and Matteson re-examined the pattern of AC topography in the zygomatic process from a radiological point of view and emphasized the restriction of the pneumatization to the temporal part of the process. They also confirmed the integrity of the process’ shape as a defining feature of the variant [62]. The term ‘defect’ was probably introduced to describe the striking radiotranslucency of the lesion on panoramic radiographs. This term calls on the association of a pathological finding and thus appears to be a misnomer what is just a variant of bone anatomy. Prior to the first study of ZAC prevalence, Roser et al. [53] and Kulikowski et al. [37] already had reported on this variant – as it can be seen on panoramic radiographs – and associated surgical implications in TMJ surgery. Furthermore, the first prevalence study on this subject based on panoramic radiographs was published prior to the reference paper of Tyndall and Matteson [1]. This publication showed a ZAC prevalence almost identical to the result published by Tyndall and Matteson one year later. However, what is now called ZAC has long been known in the otological literature as an osseous variant of variable size that requires diagnostic awareness, in particular the spread of temporal bone infections to the zygomatic arch [18].

Lang stressed the anatomical finding [39] that AC of the temporal bone’s pneumatic spaces do not respect osseous boundaries, just like other paranasal sinuses [15]. However, within the limits of the presented study, expansion of ZAC is confined to the temporal part of the zygomatic arch confirming the original description of Tremble [61]. However, Levenson et al. [40] reported a case of extracranial pneumatocele with a herniated AC of the mastoid. In this case, the pneumatization of the mastoid reached out to the root of the zygomatic arch. The intact anterior part of the zygomatic arch was bowed outward by the herniated mastoidal AC.

Since the first prevalence study of ZAC on panoramic radiographs, numerous radiological descriptions of the variant were published using this radiographic projection, initially as case reports [11], [41], [51], [63], [71], and later as large series (Table 3 (Tab. 3)). These studies describe ZAC as an incidental finding that was assumed to occur only after puberty [28]. Indeed, ZAC is an asymptomatic incidental finding [62]. However, one report detailed in a female with a history of rheumatoid arthritis and chronic facial pain the synchronous finding of ZAC ipsilateral to the trigger point of pain attacks. This patient was successfully treated with anti-inflammatory medication. The authors explicitly addressed the coincidence of the findings and denied a causal relationship between the ZAC and pain development [58]. ZAC on panoramic radiographs is usually diagnosed in less than 5% of the respective study group. For example, Yavuz et al. describe the prevalence of ZAC on panoramic radiographs in a very large group (N=8,107) of patients investigated in a dental clinic, but the prevalence was 1.03% and relatively low. Thus, variants of ZAC prevalence within a small range appear to be normal [70]. Prior to the study of Yavuz et al. [70], Hofmann et al. [27] identified the first ZAC in a seven-year old boy. This result was confirmed by others [16], [47] and these findings disproved the thesis that ZAC develop only after the puberty [28]. Indeed, ZAC prevalence in children from the age of 9 years do not differ from that in adults [16] and the present CT study revealed ZAC in children younger than presently reported. Recently, the ability of panoramic radiography was denied to describe accurately the glenoid fossa and zygomatic process pneumatization [9]. According to these authors CBCT scans would be a more appropriate choice due to their three-dimensional imaging and better spatial resolution of the region of interest. Overall, they demonstrate in their study that the prevalence of AC in panoramic radiographs (1–3.5%) was significantly lower as opposed to those by CBCT (~ 8%) [9]. 

In the present CT-based study a trend shows that with a yearly age increase the probability of ZAC decreases by approximately 1%. This would raise the question whether an age dependent bone remodeling process leads to the fact that the pneumatization is a rare finding in aged individuals. However, ZAC occur even in very old people (Figure 8 (Fig. 8)).

So far, throughout the reviewed literature there was no study on the prevalence of ZAC based on CT scans that was not taken from a dental clinic. This selection could have an impact on the frequency of ZAC due to the probability of a higher frequency of patients subjected for radiological investigations of the facial skeleton and skull base for diseases related to this region. Therefore, one aim of our study was to display the prevalence of ZAC using a less biased population. The presented data are in line with previous CT-based reports on ZAC prevalence [4], [20], [38], [43].

The numerically extensive investigation justifies the presented results. However, due to the selection of a high number of patients with adequate CT images, it could not be avoided to use pictures from the X-ray archive that had been generated by different CT scanners. In the study of Ladeira et al. [38] the images were recorded with cone beam CT or with a multislice CT. It made it no difference to use images from different sources to identify ZAC. Indeed, the prevalence of ZAC on CT is almost identical to CB-CT [43]. With reference to these reports it can be assumed for the present study that the use of two different multislice CT scanners does not substantially affect the imaging capability of ZAC. Ladeira’s et al. study and others [20], [38] confirm that prevalence of pneumatization based on images derived from three-dimensional techniques is higher than by panoramic radiographs. Nevertheless, the presented review on ZAC radiology does not support the generalization of these authors’ statements as far as the radiological identification of ZAC is concerned (Table 3 (Tab. 3) and Table 4 (Tab. 4)). 

However, an important parameter for the evaluation of very fine air spaces could be the layer thickness at which the images were evaluated. In this work it was found with a relatively coarse layer thickness of 3 mm a still not very different prevalence of 7.57%. So far, Bronoosh et al. [4] used the thinnest layer (layer thickness = 0.625 mm) and published a slightly higher prevalence of ZAC (9.55%). Groell and Fleischmann [20] used a layer thickness of 1–2 mm and reported a prevalence of 5%. The work of Ladeira et al. [38] has the highest prevalence with 21.3%, however, in this work, unfortunately no information on the layer thickness could be found. In summary, the impact of layer thickness of cross sectional images apparently does not affect the imaging of ZAC. One advantage using CT is that more detailed analyzes can be done [52]. For example the volume of temporal bone pneumatization can be determined using 3D reconstructions [24], [30].

Ladeira et al. [38] pointed to the probability of laterality in ZAC. Unilateral ZAC occurred on the left side in 60.5% in their study. Left sided unilateral ZAC were more frequently in the present study, but the data are presently not conclusive to definitely address the potential laterality of ZAC.

One study examined the correlation between ZAC and the degree of pneumatization of the remaining regions of the temporal bone [4]. It was stated that once ZAC is present, the degree of pneumatization in the rest of the temporal bone is significantly higher. We did not investigate the temporal bone pneumatization in general, but interpretation of data should consider the high variability of temporal bone pneumatization in human beings and the temporo-spatial effect of external factors on this phenomenon [68].

Radiotranslucent lesions of the zygomatic arch other than ZAC are very rare but have to be considered: haemangioma [8], [31], aneurysmal bone cyst [13], eosinophilic granuloma [19], [23], and metastasis [42]. Furthermore, extensive pneumatization of the sphenoid sinus can superimpose the zygomatic arch on panoramic radiographs [2]. ZAC can become involved in the spread of atypical mastoiditis to other pneumatized areas of temporal bone. Indeed, inflammation of the TMJ can be the result of mastoiditis [14]. On the other hand, a TMJ inflammation may cause an otitis externa [60]. Both ways of infection transmission do not require ZAC. TMJ surgery has to consider possible structural changes of the zygomatic arch [5], [26], [35], [37], [44], [51], [64]. 

Presently, magnetic resonance imaging is not well established in ZAC diagnosis. Low signals overlying the glenoid fossa were attributed to extensive temporal bone pneumatization impairing TMJ diagnostics [69]. However, Randzio et al. [51] showed pneumatization of the zygomatic arch could be distinguished from joint findings on sagittal sections of MRI.

## Conclusion

ZAC is a relatively frequent anatomical variant of the lateral skull base that needs no treatment. Knowledge about this variant is mandatory in TMJ surgery planning, malar surgery including the distal zygomatic arch, and also in the assessment of topographically related pathologies. ZAC on CT is a distinct lesion within a zygomatic arch that shows no alteration of the bone’s shape. ZAC is a homogeneously radio-translucent lesion. Attention should be paid to alterations of this radiological appearance. Presently, the literature does not provide convincing evidence in favour for sectional imaging to identify ZAC more frequently than do panoramic radiographs. However, the internal structure of ZAC and communication to the air cell system of the temporal bone can only be studied on adequate sectional images.

## Notes

### Competing interests

The authors declare that they have no competing interests.

### Authorship

The authors REF and LV contributed equally to this publication.

## Figures and Tables

**Table 1:**
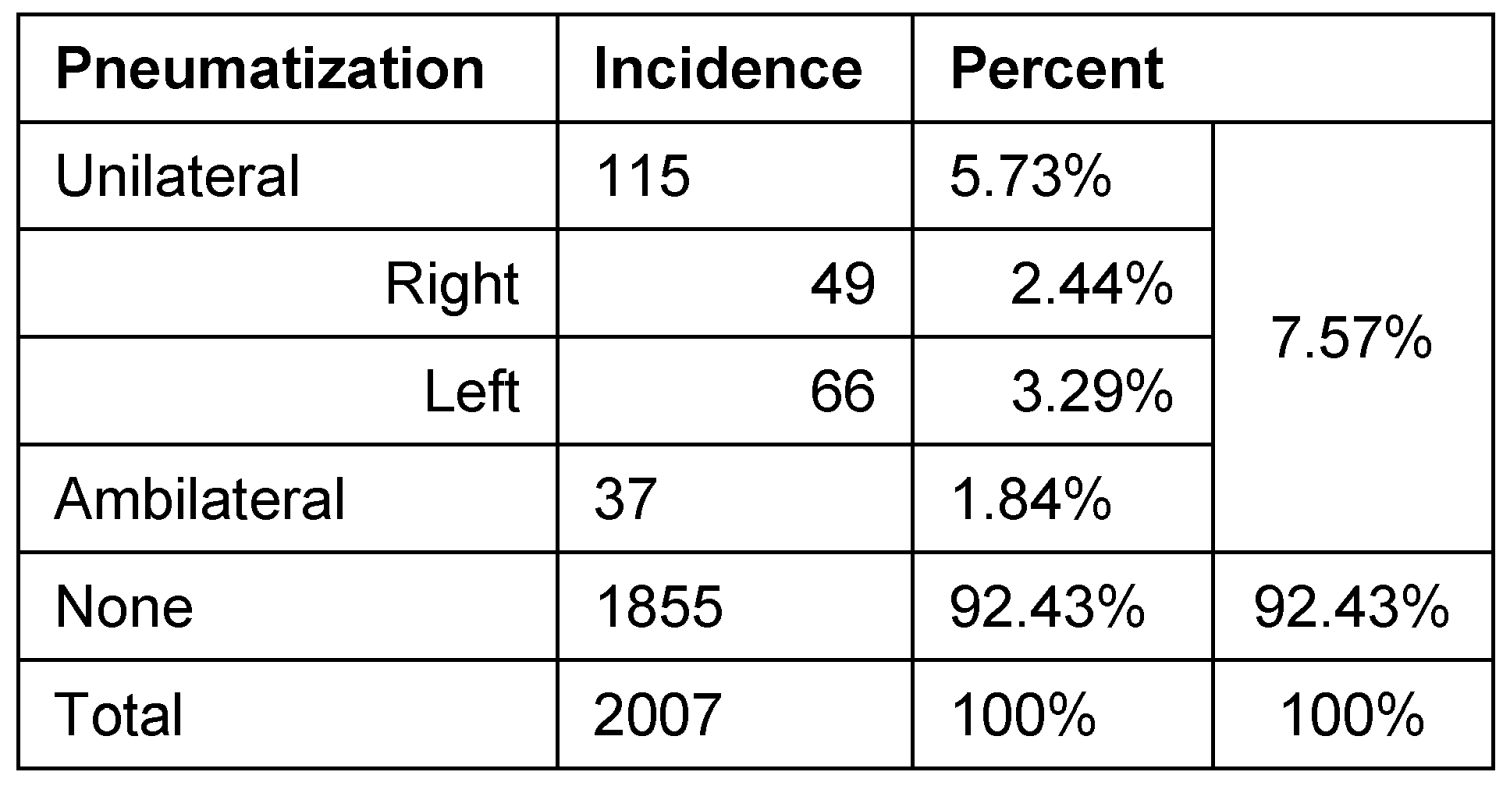
Localization of pneumatization

**Table 2:**
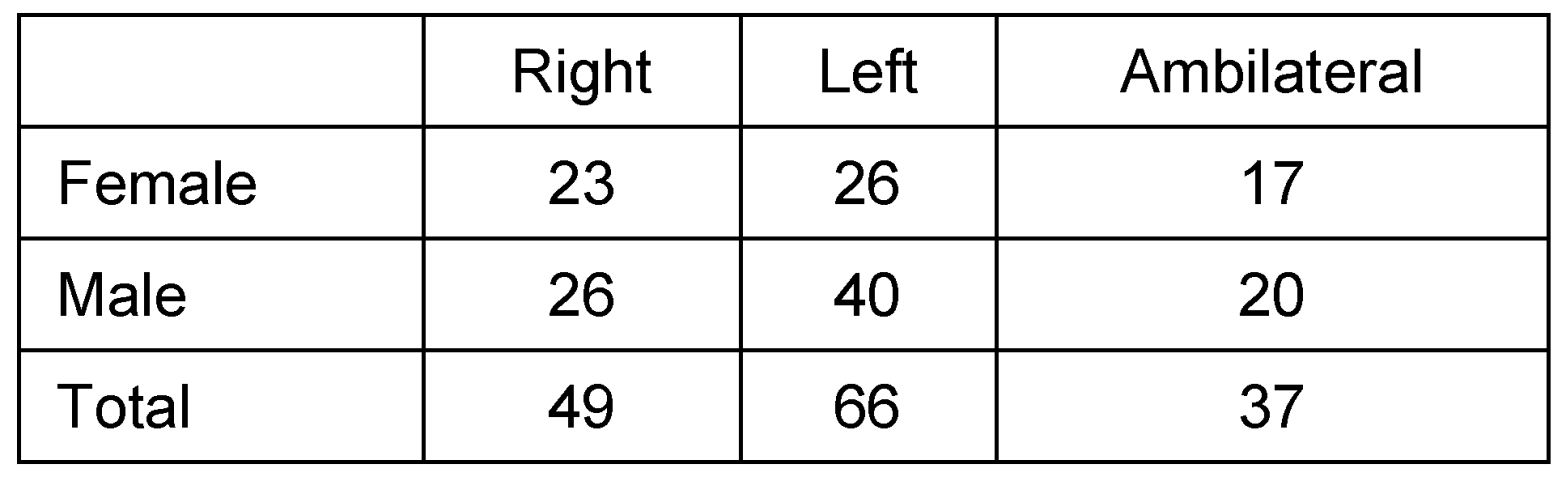
Localization of pneumatization depending on laterality and gender

**Table 3:**
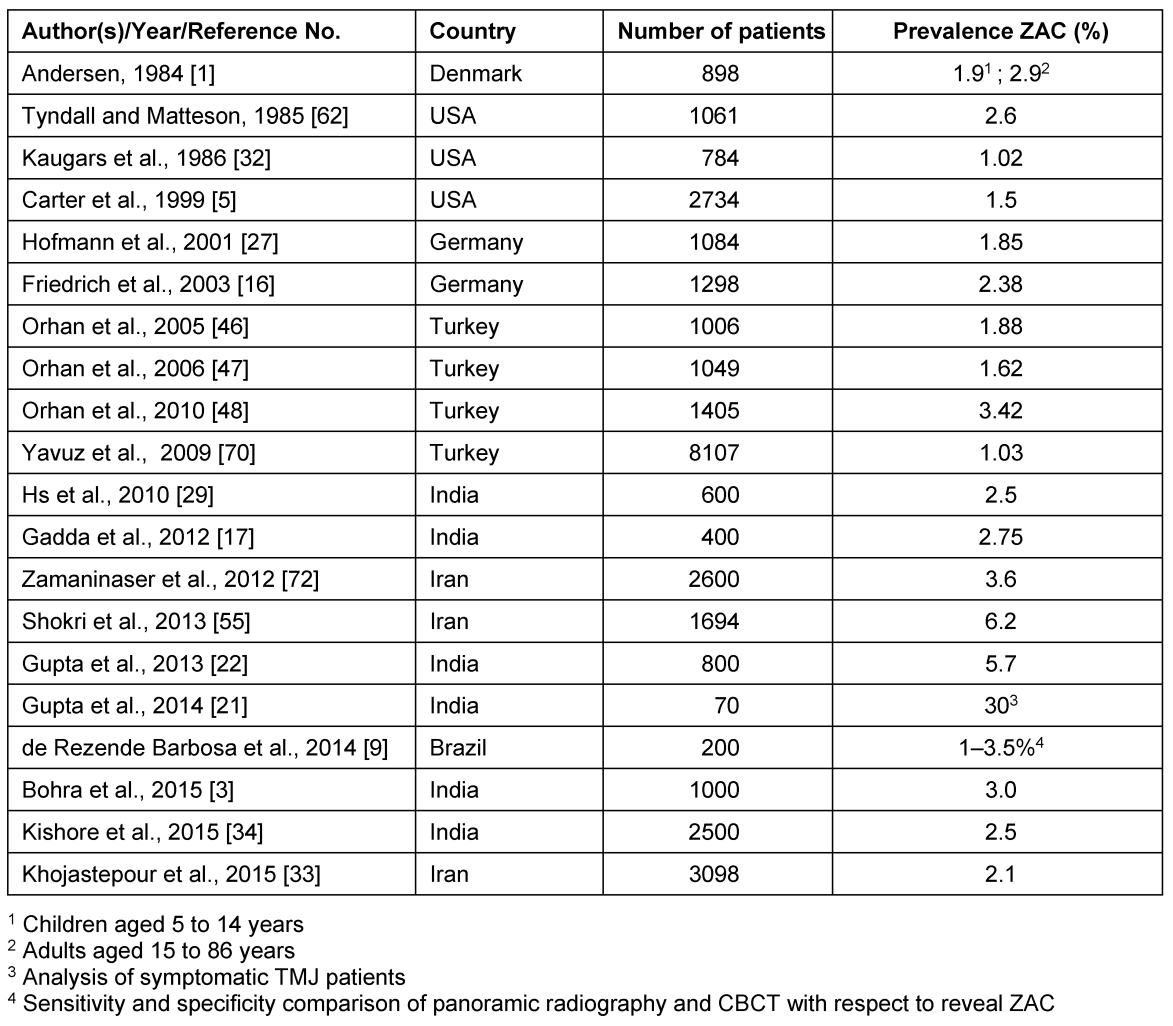
Studies on ZAC performed with panoramic radiographs

**Table 4:**
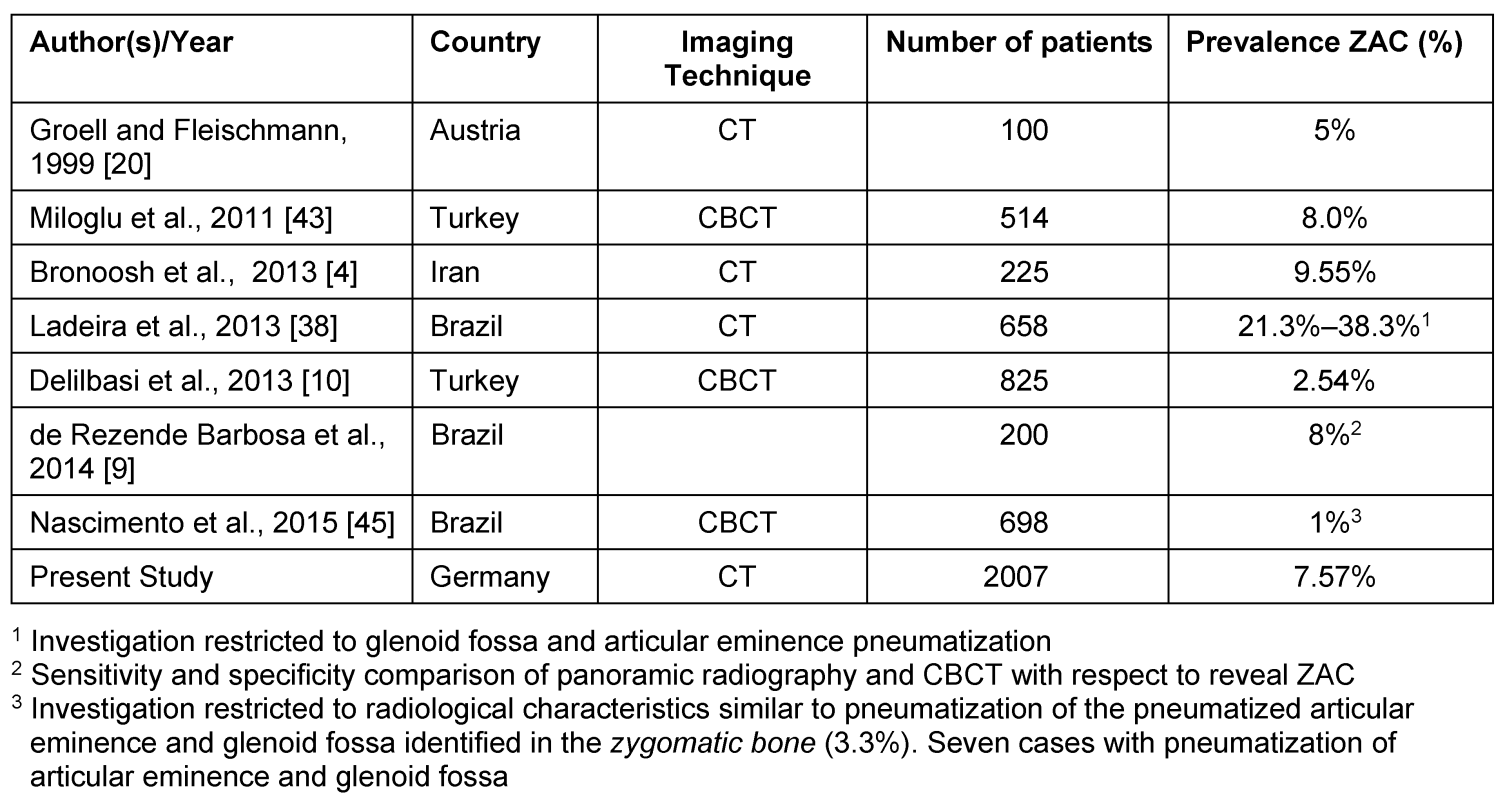
Studies on ZAC performed with computed tomography or CBCT

## References

[1] Andersen V (1984). Frekvensen af cystelignende pneumatisering af tuberculum articulare på panoramatomogrammer. Tandlaegebladet.

[2] Bishop MG, Smith NJ (1987). Pneumatisation of the pterygoid plates of the sphenoid bone as a normal finding on dental panoramic tomographs. Br Dent J.

[3] Bohra A, Bhagwati BT, Patil N, Anitha B, Bhateja S (2015). Prevalence of zygomatic air cell defect: a panoramic radiographic study. Indian J Dent Adv.

[4] Bronoosh P, Shakibafard A, Mokhtare MR, Munesi Rad T (2014). Temporal bone pneumatisation: a computed tomography study of pneumatized articular tubercle. Clin Radiol.

[5] Carter LC, Haller AD, Calamel AD, Pfaffenbach AC (1999). Zygomatic air cell defect (ZACD). Prevalence and characteristics in a dental clinic outpatient population. Dentomaxillofac Radiol.

[6] Chen T, Hsu Y, Li J, Hu J, Khadka A, Wang Q, Wang D (2011). Correction of zygoma and zygomatic arch protrusion in East Asian individuals. Oral Surg Oral Med Oral Pathol Oral Radiol Endod.

[7] da Costa Ribeiro R, dos santos BJ, Provenzano N, de Freitas PH (2014). Dautrey's procedure: an alternative for the treatment of recurrent mandibular dislocation in patients with pneumatization of the articular eminence. Int J Oral Maxillofac Surg.

[8] Davis E, Morgan LR (1974). Hemangioma of bone. Arch Otolaryngol.

[9] de Rezende Barbosa GL, Nascimento Mdo C, Ladeira DB, Bomtorim VV, da Cruz AD, Almeida SM (2014). Accuracy of digital panoramic radiography in the diagnosis of temporal bone pneumatization: a study in vivo using cone-beam-computed tomography. J Craniomaxillofac Surg.

[10] Delilbasi C, Orhan K, Icen M, Aksoy S, Horasan S, Kenan Kose S (2013). Evaluation of articular eminence pneumatization using cone beam computed tomography. Minerva Stomatol.

[11] Deluke DM (1995). Pneumatization of the articular eminence of the temporal bone. Oral Surg Oral Med Oral Pathol Oral Radiol Endod.

[12] Diamant M (1954). Size variation of the mastoid air cell system according to Wittmaack, Schwarz and Diamant. Acta Otolaryngol Suppl.

[13] Eveson JW, Moos KF, MacDonald DG (1978). Aneurysmal bone cyst of the zygomatic arch. Br J Oral Surg.

[14] Faerber TH, Ennis RL, Allen GA (1990). Temporomandibular joint ankylosis following mastoiditis: report of a case. J Oral Maxillofac Surg.

[15] Fraederich M, Aboelhasan MF, Knips J, Heiland M, Friedrich RE (2015). Nasal speech associated with hyperaeration of the sphenoid sinus. In Vivo.

[16] Friedrich RE, Scheuer HA, Scheuer J (2003). Pneumatisationen des Jochbogens ('Zygomatic Air Cell Defect') auf Panoramaschichtaufnahmen von Kindern und Jugendlichen als Hilfsmittel zur Identitätsbestimmung und Altersschätzung. Rechtsmedizin.

[17] Gadda R, Patil NA, Salvi R (2012). Zygomatic air cell defect: prevalence and characteristics in dental outpatient population. J Contemp Dent.

[18] Gadolin HR (1938). Beiträge zur Pathologie und Klinik der sog. Zygomatikomastoiditis. Acta Otolaryngol.

[19] Ginsberg HN, Swayne LC, Peron DL, Magidson JG, Newcomb AW (1988). Bilateral temporal bone involvement with eosinophilic granuloma. Comput Med Imaging Graph.

[20] Groell R, Fleischmann B (1999). The pneumatic spaces of the temporal bone: relationship to the temporomandibular joint. Dentomaxillofac Radiol.

[21] Gupta D, Rashmi NC, Sheikh S, Pallagatti S, Goyal G, Singh R, Parnami P, Singh G (2014). The prevalence, radiographic appearance, and characteristics of zygomatic air cell defects (ZACDs) in symptomatic temporomandibular joint disorder patients in North Indian population. Oral Maxillofac Surg.

[22] Gupta D, Sheikh S, Pallagatti S, Aggarwal A, Goyal G, Chidanandappa RN, Parnami P (2013). Zygomatic air cell defect: a panoramic radiographic study of a North Indian population. J Investig Clin Dent.

[23] Hadjigeorgi C, Parpounas C, Zarmakoupis P, Lafoyianni S (1990). Eosinophilic granuloma of the temporal bone: radiological approach in the pediatric patient. Pediatr Radiol.

[24] Han SJ, Song MH, Kim J, Lee WS, Lee HK (2007). Classification of temporal bone pneumatization based on sigmoid sinus using computed tomography. Clin Radiol.

[25] Hasnaini M, Ng SY (2000). Extensive temporal bone pneumatization: incidental finding in a patient with TMJ dysfunction. Dent Update.

[26] Helman J, Laufer D, Minkov B, Gutman D (1984). Eminectomy as surgical treatment for chronic mandibular dislocations. Int J Oral Surg.

[27] Hofmann T, Friedrich RE, Wedl JS, Schmelzle R (2001). Pneumatisation des Jochbogens auf Panoramaschichtaufnahmen. Mund Kiefer Gesichtschir.

[28] Hollinshead WH (1968). Anatomy for surgeons.

[29] Hs S, Patil K, Vg M (2010). Zygomatic air cell defect: A panoramic radiographic study of a south Indian population. Indian J Radiol Imaging.

[30] Jadhav AB, Fellows D, Hand AR, Tadinada A, Lurie AG (2014). Classification and volumetric analysis of temporal bone pneumatization using cone beam computed tomography. Oral Surg Oral Med Oral Pathol Oral Radiol.

[31] Jeter TS, Hackney FL, Aufdemorte TB (1990). Cavernous hemangioma of the zygoma: report of cases. J Oral Maxillofac Surg.

[32] Kaugars GE, Mercuri LG, Laskin DM (1986). Pneumatization of the articular eminence of the temporal bone: prevalence, development, and surgical treatment. J Am Dent Assoc.

[33] Khojastepour L, Mirbeigi S, Ezoddini F, Zeighami N (2015). Pneumatized Articular Eminence and Assessment of Its Prevalence and Features on Panoramic Radiographs. J Dent (Tehran).

[34] Kishore M, Panat SR, Kishore A, Aggarwal A, Upadhyay N, Agarwal N (2015). Prevalence of Zygomatic Air Cell Defect using Orthopantomogram. J Clin Diagn Res.

[35] Kraut RA (1985). Methyl methacrylate obturation of the pneumatized articular eminence of the temporal bone. J Oral Maxillofac Surg.

[36] Kuczkowski J, Narozny W, Stankiewicz C, Mikaszewski B, Izycka-Swieszewska E (2005). Zygomatic abscess with temporal myositis - a rare extracranial complication of acute otitis media. Int J Pediatr Otorhinolaryngol.

[37] Kulikowski BM, Schow SR, Kraut RA (1982). Surgical management of a pneumatized articular eminence of the temporal bone. J Oral Maxillofac Surg.

[38] Ladeira DB, Barbosa GL, Nascimento MC, Cruz AD, Freitas DQ, Almeida SM (2013). Prevalence and characteristics of pneumatization of the temporal bone evaluated by cone beam computed tomography. Int J Oral Maxillofac Surg.

[39] Lang J (1992). Klinische Anatomie des Ohres.

[40] Levenson MJ, Ahuja G, Bergeron T (1989). Spontaneous extracranial pneumatocele associated with mastoid hyperpneumatization. Arch Otolaryngol Head Neck Surg.

[41] Lindenmuth JE, Clark MS (1986). Pneumatization of the articular eminence. Cranio.

[42] Mennie JC, Reid R, Cowie F, Hilmi O (2011). Ewing's Sarcoma of the Zygomatic Arch Presenting in a 69-Year Old: An Unusual Case Report. Case Rep Otolaryngol.

[43] Miloglu O, Yilmaz AB, Yildirim E, Akgul HM (2011). Pneumatization of the articular eminence on cone beam computed tomography: prevalence, characteristics and a review of the literature. Dentomaxillofac Radiol.

[44] Mizuno A, Suzuki S, Motegi K (1988). Articular eminectomy for long-standing luxation of the mandible. Report of 2 cases. Int J Oral Maxillofac Surg.

[45] Nascimento HA, Visconti MA, Macedo Pde T, Haiter-Neto F, Freitas DQ (2015). Evaluation of the zygomatic bone by cone beam computed tomography. Surg Radiol Anat.

[46] Orhan K, Delilbasi C, Cebeci I, Paksoy C (2005). Prevalence and variations of pneumatized articular eminence: a study from Turkey. Oral Surg Oral Med Oral Pathol Oral Radiol Endod.

[47] Orhan K, Delilbasi C, Orhan AI (2006). Radiographic evaluation of pneumatized articular eminence in a group of Turkish children. Dentomaxillofac Radiol.

[48] Orhan K, Oz U, Ulas O, Orhan AI, Ulker AE, Delilbasi C, Akcam O (2010). Investigation of pneumatized articular eminence in orthodontic malocclusions. Orthod Craniofac Res.

[49] Piette E (1986). La pneumatisation du tubercule zygomatique antérieur de l'os temporal. Rev Stomatol Chir Maxillofac.

[50] Puelacher WC, Waldhart E (1993). Miniplate eminoplasty: a new surgical treatment for TMJ-dislocation. J Craniomaxillofac Surg.

[51] Randzio J, Vogl T, Kellermann O, Geisemeyer W (1989). Zur Pneumatisation des Tuberculum articulare des Kiefergelenkes. Eine Fallbeschreibung. Dtsch Z Mund Kiefer Gesichtschir.

[52] Romano-Sousa CM, Garritano-Papa E (2015). Pneumatization of the temporal portion of the zygomatic arch: The contribution of computed tomography to the reconstruction in volumetric two-dimensional and three-dimensional, with the aid of image rendering protocols. Indian J Dent Res.

[53] Roser SM, Rudin DE, Brady FA (1976). Unusual bony lesion of the zygomatic arch. J Oral Med.

[54] Seppälä AJ (1946). Variations in the pneumatic cell system of the temporal bone; acute otitis media and its complications in varying types of temporal pneumatization. Acta Oto Laryngol.

[55] Shokri A, Noruzi-Gangachin M, Baharvand M, Mortazavi H (2013). Prevalence and characteristics of pneumatized articular tubercle: First large series in Iranian people. Imaging Sci Dent.

[56] Srivathsa SH, Guledgud MV, Patil K (2012). Zygomatic air cell defect. J Cranio Max Dis.

[57] Stewart JP (1928). The Histopathology of Mastoiditis. Proc R Soc Med.

[58] Stoopler ET, Pinto A, Stanton DC, Mupparapu M, Sollecito TP (2003). Extensive pneumatization of the temporal bone and articular eminence: an incidental finding in a patient with facial pain. Case report and review of literature. Quintessence Int.

[59] Tang K, Hsu Y, Hu J, Zhao Y, Wang Q, Li J (2014). New horizontal v-shaped osteotomy for correction of protrusion of the zygoma and the zygomatic arch in East Asians: indication and results. Br J Oral Maxillofac Surg.

[60] Thomson HG (1989). Septic arthritis of the temporomandibular joint complicating otitis externa. J Laryngol Otol.

[61] Tremble GE (1934). Pneumatization of the temporal bone. Arch Otolaryngol.

[62] Tyndall DA, Matteson SR (1985). Radiographic appearance and population distribution of the pneumatized articular eminence of the temporal bone. J Oral Maxillofac Surg.

[63] Tyndall DA, Matteson SR (1987). The zygomatic air cell defect (ZACD) on panoramic radiographs. Oral Surg Oral Med Oral Pathol.

[64] Undt G, Rasse M (1996). Die chirurgische Therapie der rezidivierenden kondylären Luxation des Kiefergelenks. Acta Chir Austria.

[65] Virapongse C, Sarwar M, Bhimani S, Sasaki C, Shapiro R (1985). Computed tomography of temporal bone pneumatization: 1. Normal pattern and morphology. AJR Am J Roentgenol.

[66] Warnaar A, Snoep G, Stals FS (1989). A swollen cheek, an unusual course of acute mastoiditis. Int J Pediatr Otorhinolaryngol.

[67] Weinberg S (1984). Eminectomy and meniscorhaphy for internal derangements of the temporomandibular joint. Rationale and operative technique. Oral Surg Oral Med Oral Pathol.

[68] Wittmaack K (1918). Über die normale und pathologische Pneumatisation des Schläfenbeins.

[69] Wong K, Munk PL (1999). Magnetic resonance imaging of the temporomandibular joint: diagnostic difficulty caused by extensive pneumatization of the mastoid air cells. Skeletal Radiol.

[70] Yavuz MS, Aras MH, Güngör H, Büyükkurt MC (2009). Prevalence of the pneumatized articular eminence in the temporal bone. J Craniomaxillofac Surg.

[71] Yurosko JJ (1985). Pneumatized articular eminence. Oral Surg Oral Med Oral Pathol.

[72] Zamaninaser A, Rashidipoor R, Mosavat F, Ahmadi A (2012). Prevalence of zygomatic air cell defect: Panoramic radiographic study of a selected Esfehanian population. Dent Res J (Isfahan).

